# Building policy-making capacity in the Ministry of Health: the Kazakhstan experience

**DOI:** 10.1186/1478-4491-13-4

**Published:** 2015-01-20

**Authors:** Tata Chanturidze, Orvill Adams, Bolat Tokezhanov, Mike Naylor, Erica Richardson

**Affiliations:** Oxford Policy Management Ltd, 6 St Aldates Courtyard, 38 St Aldates, Oxford, OX1 1BN UK; Ministry of Health, Kazakhstan, 8, Orinbor Street, Ministry house, 5th Entrance, 010000 Astana, Kazakhstan; European Observatory on Health Systems and Policies, London School of Hygiene and Tropical Medicine, 15-17 Tavistock Place, London, WC1H 9SH UK

**Keywords:** Capacity-building, Strategic planning, Kazakhstan, Competence framework, Needs assessment

## Abstract

**Background:**

Recent economic growth in Kazakhstan has been accompanied by slower improvements in population health and this has renewed impetus for health system reform. Strengthening strategic planning and policy-making capacity in the Ministry of Health has been identified as an important priority, particularly as the Ministry of Health is leading the health system reform process.

**Case description:**

The intervention was informed by the United Nations Development Programme (UNDP) framework for capacity building which views capacity building as an ongoing process embedded in local institutions and practices. In response to local needs extra elements were included in the framework to tailor the capacity building programme according to the existing policy and budget cycles and respective competence requirements, and link it with transparent career development structures of the Ministry of Health. This aspect of the programme was informed by the institutional capability assessment model used by the United Kingdom National Health Service (NHS) which was adapted to examine the specific organizational and individual competences of the Ministry of Health in Kazakhstan.

**Discussion and evaluation:**

There were clear successes in building capacity for policy making and strategic planning within the Ministry of Health in Kazakhstan, including better planned, more timely and in-depth responses to policy assignments. Embedding career development as a part of this process was more challenging. This case study highlights the importance of strong political will and high level support for capacity building in ensuring the sustainability of programmes. It also shows that capacity-building programmes need to ensure full engagement with all local stakeholders, or where this is not possible, programmes need to be targeted narrowly to those stakeholders who will benefit most, for the greatest impact to be achieved. In sum, high quality tailor-made capacity development programmes should be based on thorough needs assessment of individual and organizational competences in a specific institutional setting.

**Conclusions:**

The experience showed that complementary approaches to human resource development worked effectively in the context of organizations and systems, where an enabling environment was present, and country ownership and political will was complemented by strong technical assistance to design and deliver high quality tailor-made capacity building initiatives.

## Background

Kazakhstan is an oil-rich, upper-middle-income country in central Asia which has experienced strong economic growth since the year 2000, with annual gross domestic product (GDP) growth reaching 6% in 2013 despite falling oil prices [1]. This prosperity has been shared broadly and the share of the country’s population of 16.9 million who live below the national poverty line has fallen rapidly, from 46.7% in 2001 to 2.9% in 2013 [2]. However, Kazakhstan’s health outcomes have lagged behind its booming economy and it had one of the shortest average life expectancies in the WHO European Region at 68.6 years (63.7 years for men and 73.5 years for women) in 2010 [2], and healthy life expectancy was just 58.2 years [3]. According to estimates, infant mortality has been falling (from 38.1 per 1000 live births in 2000 to 16.7 in 2012), as has maternal mortality (from 70 per 100 000 live births in 2000 to 51 in 2012), but these health indicators remain high by European standards [2].

However, health has moved up the policy agenda in Kazakhstan and there is renewed impetus for restructuring the healthcare system. The restructuring process is wide-ranging as it encompasses wholesale reform of health financing mechanisms and provider payment mechanisms to improve efficiency and equity in the system, alongside reforms to the health care delivery system to strengthen primary, secondary and tertiary care [4]. Healthcare financing reforms include the recentralization of financial pooling arrangements and the reintroduction of provider payments mechanisms using diagnosis-related groups (DRGs) [4]. These ambitious plans seek to improve population health and have highlighted the need to strengthen managerial capacity in the health system as a whole.

The key actor in governing the health system is the Ministry of Health which is responsible for strategic planning and policy-making, but historically such capacity has been quite weak. When Kazakhstan gained independence from the Soviet Union in 1991, the inherited government structures were organized according to an administrative-command model which was designed to process orders from central authorities in Moscow. Republican-level administrators were able to influence policy, but most strategic planning decisions were also taken centrally and implemented through the vertical hierarchy. Management and budgeting decisions were, therefore, not made and implemented according to locally determined needs but by centrally determined norms and procedures, and civil servants were not well practiced in strategic planning, policy-making or budgeting [5]. Such capacity has developed out of necessity since 1991, when the country gained independence and the Ministry of Health had to act autonomously, but it was not the focus of reform efforts until more recently.

In this paper we first describe the needs assessment approach used in the capacity building programme by breaking it down into the eight steps taken: i) identifying key stakeholders; ii) engaging stakeholders; iii) tailoring the tools; iv) assessing capacity needs; v) designing the response; vi) implementing the response; vii) evaluating the response; viii) creating enabling environments for career development. The outcomes of these steps are then discussed so that in the conclusions we are able to give the key lessons from the implementation and evaluation processes which could inform national and international actors working on similar projects elsewhere in the world.

## Case description

### Needs assessment approach

In joint work with the Ministry of Health it was agreed that a United Nations Development Programme (UNDP) approach to the capacity development process would provide the most useful starting point as it has been used successfully elsewhere in the region [6]. The UNDP approach to capacity development is a well-established model for transnational working which can be used flexibly to fit country-specific needs and ‘buy-in’ from in-country policy-makers is considered best practice. This approach outlines five key steps for capacity building: (i) engaging stakeholders in capacity development; (ii) assessing capacity needs and assets; (iii) formulating capacity development response; (iv) implementing a capacity development response; and (v) evaluating capacity development [6]. However, the approach required adapting to the specific context in Kazakhstan, so it was agreed that additional steps should be included, to make an eight stage process that would identify missing policy-making competencies within the Ministry of Health, and tailor the competence assessment tools.

**Step 1: Identifying key stakeholders:** Which departments are involved in policy development and strategic planning at the Ministry of Health? How is Ministry of Health policy-making linked to the work of other Ministries and national policy-making processes, and which departments participate?

**Step 2: Engaging stakeholders**: What is the stimulus for these departments and individual staff members to be engaged in the capacity building programme? How can they be further incentivized?

**Step 3: Tailoring tools for competence needs assessment**: What competencies are required to undertake policy analysis and strategy development functions in the Ministry of Health? Which areas of technical competence deserve the most attention?

**Step 4: Assessing capacity needs**: What methods are to be most relevant for assessing the organizational capability and the individual competencies necessary for policy making at the Ministry of Health?

**Step 5: Designing capacity development response**: How can the capacity-building programme be most effective? What methods of capacity building are most suitable to address the big knowledge gaps? How can mentoring and coaching be best tailored and undertaken?

**Step 6: Implementing a capacity development response**: How to achieve maximum quality and effectiveness in the delivery of the capacity-building programme?

**Step 7: Evaluating capacity development results**: What are the most suitable methods for evaluating the Ministry of Health capacity-building programme outputs and outcomes? How can the evaluation results be used for incentivizing and promoting personnel?

**Step 8: Creating enabling environment for career development**: What are the features of an enabling environment for career development based on upgraded competencies? How can these be put in place in the given context?

These extra steps placed a particular emphasis on developing an understanding and acceptance of competencies by staff. Traditionally, capacity building in the Ministry of Health had focussed on increasing the skills of individuals. That is, providing individuals with abilities or expertise in specific activities or tasks whereas competencies incorporate skills but also include abilities and behaviours, as well as knowledge that is fundamental to the use of skills. The Ministerial leadership agreed that capacity building should focus on competencies with a view to providing staff with an understanding of their jobs and how to improve performance. Staff were thereby encouraged to be more flexible and to use their cumulative skills and knowledge to solve problems.

### Identifying the key stakeholders

The key stakeholders were identified through a mapping exercise according to their current engagement in the policy cycle or, where gaps were identified, where it was required. Most stakeholders were identified in the Ministry of Health – of the five departments under the Ministry, the Department of Strategy Development (DSD) had the main responsibility for handling policy formation and developing medium- and long-term strategies and the staff worked closely with the Department for Economy and Finance (DEF) which provided the necessary financing and budgeting input. The Department for Organization of Medical Care, Department of Science and Human Resources and the Department of Administrative and Legal Work were found to be peripheral to the annual policy, planning and budgeting cycle for the Ministry of Health. Outside the Ministry of Health, stakeholders were identified in health departments at the regional level, where strategic plans are developed in relation to the needs of the local population. The Ministry of Health also has formal and regular consultations with other ministries and departments, but the key contacts were with the Ministry of Finance, the Ministry of Economic Development and Trade, and the Ministry of Justice.

The mapping exercise identified only a handful of individuals in departments within the Ministry of Health who were proactively engaged in the policy development and strategic planning process. The DSD was central to this role and consisted of four divisions with a total of fourteen staff members – four heads of division, four chief experts and six experts. These were the key stakeholders who were initially targeted in the capacity building programme, although the lack of continuity hampered progress (see below).

Policy-makers wanted to embark on a broader programme to build capacity across the Ministry of Health rather than focus on those departments which had existing capacity which could be developed further. To promote ownership, three departments (of the five – not just DSD) were selected on the basis of both strategic importance and existing engagement in policy-making for inclusion in the capacity-building programme rather than focusing efforts more narrowly. However, in the final analysis, the ease with which departments could be engaged in the process reflected their existing engagement with policy development and strategic planning rather than their strategic importance, as they had already demonstrated an interest and willingness to engage in these activities. By the end of the programme, 17 modules were designed and delivered on a wide range of relevant topics with 10 to 15 participants enrolled in each module and three study tours on DRGs in Switzerland were designed and delivered, with 8 to 15 participants in each study tour.

### Engaging stakeholders

In order to understand the incentives required to engage both individuals and departments in the capacity building programme, we examined which stimuli needed to be in place by asking the stakeholders, specifically:

-  Why should these departments engage in the capacity building programme?
-  Why should individual staff members engage in the programme?
-  If incentives are not currently in place, can they be introduced?

We found that individuals were incentivized around the following factors:

-  Enhanced professional image;
-  Gaining confidence and greater ability to participate in discussions and disputes in their specific technical area;
-  Moving to a more comfortable environment;
-  Reaching a position where promotion could be requested or granted.

These factors were then built into the capacity building programme and participants were informed of how the programme can contribute to their professional and personal development. This background work identifying the potential benefits of the capacity building programme to the selected departments of the Ministry of Health also identified the need for more knowledge about the functions and competencies needed for policy development and strategic planning to build on existing capacity assets. The approach agreed was to:

-  Define the areas for organizational and individual competence development;
-  Involve departments in the comprehensive capacity building programme, combining trainings, study tours, coaching, mentoring and doing by learning activities;
-  Increase possibilities for improving the working environment;
-  Identify merit-based career enhancement possibilities;
-  Increase the scope for meaningful promotion based on increased competencies and improved performance.

### Tailoring tools for competence needs assessment

In order to assess adequately the capacity needs and assets in the Kazakh Ministry of Health, it was first necessary to map the Ministry of Health policy-making functions around the policy and budget cycles. This entailed identifying the specific functions and processes that were already present in the Ministry of Health, and whether these systems and processes allowed for transparent and evidence-based policy making [7]. Namely, we mapped whether the Ministry of Health had operationalized and institutionalized:

-  Policy, strategy and budget planning cycles that were interlinked, consistent and harmonized;
-  Instruments and data to support the policy making cycles, including the National Health Reports and National Health Accounts, and evidence from routine Health Monitoring and Information Systems;
-  Processes allowing engagement of various stakeholders within the Ministry of Health itself, as well as from other government structures and external partners;
-  Processes allowing the oversight, monitoring and evaluation of policy implementation;
-  Processes to allow incorporating the lessons learnt in previous policy cycles.

The institutional capability assessment model used by the United Kingdom National Health Service (NHS) was chosen as a good fit because its features could easily be adapted to examine the specific organizational and individual competences of the Ministry of Health in Kazakhstan [8]. The Institutional Capability Review is a tool to help organizations to recognize weaknesses, and put in place capacity development measures to mitigate them. The review is based on a framework to assess government ministries and their civil servants, as well as to identify areas that could be strengthened [8]. In parallel, the list of positions in the three selected departments were analysed together with individual job descriptions, to deepen understanding of existing needs for individual capacity building.

**Figure 1:**
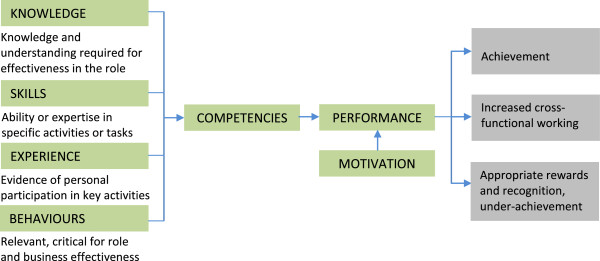
**Model of** **required roles about here.**

Performance assessment elements were then incorporated into the competence assessment: how knowledge, skills, experience and behaviours combine with competencies and lead to performance (see Figure 1). To be effective people must have a supportive working environment, the tools and instruments to do the job, appropriate direction and supervision, and motivation. In addition, the individual must have the appropriate competencies and skills to undertake the tasks required of them. The competencies assess ‘how’ staff perform, rather than just ‘what’ they do [7].

### Assessing capacity needs and assets

Once the tools and approach had been approved and agreed by Ministry of Health stakeholders and the external partners, the capacity needs and assets could be assessed. Two data collection instruments were developed in the form of written questionnaires to be completed by stakeholders. The Institutional Capability Review Instrument was developed on the basis of the NHS Model of Capability (see above) and stakeholders were invited to rate their department’s current performance in the different areas of capability (leadership, strategy and delivery) [9]. Individual capabilities were self-assessed using an Individual Competency and Skills Assessment Instrument [7, 9]. Tables 1 and 2 show the results of the institutional and individual capability assessments, respectively. Table 1 shows the assessment results for institutional capabilities broken down by area – Leadership, Strategy and Delivery – and outlines the competences covered. Table 2 shows the results of individual capability assessments with capabilities organized as to whether capability is strong or requires development. It was understood that individuals would not be inclined to recognize extreme weaknesses in their competencies for fear of losing their jobs, because they knew the capability assessment results were communicated with the management. Therefore, it was understood that the assessment results would be biased towards positive self-rating and this knowledge was taken into account when drawing conclusions and moderated the strength of recommendations made.

**Table 1:** **Results of institutional capability assessment**

Area of capability	Area of competence	Institutional capabilities, assessment results
Leadership	• Setting direction	Needs development: 11.4%
	• Igniting passion, pride and drive	Well positioned: 66.7%
	• Take responsibility for delivery and change	Strong: 21.9%
	• Develop people	Total: 100%
Strategy	• Focus on outcomes	Needs development: 4.9%
	• Decisions made on evidence and need collaboration and building common purpose	Well positioned: 61.1%
		Strong: 34.0%
		Total: 100%
Delivery	• Plan, resource and prioritizes	Needs development: 6.6%
	• Develop clear roles, responsibilities and delivery models	Well positioned: 77.0%
	• Manage performance and value for money	Strong: 16.4%
		Total: 100%

**Table 2:** **Results of individual capability assessment in three target departments**

Capable/strong capability	Needs development/weak
**Formulate policy and strategy**	
Understand policy and budget cycles and financial planning	Formulate policy and strategy
	Presenting technical data
Understand governance and legal environment	Technical report writing
Understand country development policies	Political analysis
	System’s thinking
**Leadership**	
Strategic planning	Decision making
Establishing performance goals	Leading people
	Ability to create a vision
	Set priorities and be focused
**Ability to commission**	
Define a product	Conflict resolution
Manage knowledge and undertake robust needs assessment	Monitoring and evaluation of projects
Oversight contract implementation	Organizing the process
**Policy review**	
Critical thinking	Research
Quantitative analyses	Data analysis
	Qualitative analysis
	Ability to identify special problems and frame questions for analyses and research
**Policy dialogue**	
Stakeholder and political analysis	Ability to address both politically controversial and technically complex aspects of an issue in a dispute
	Ability to bring diverse interest groups to the table
	Ability to seek to formulate practical solutions to complex problems
	Ability to moderate complex issues and build consensus recommendations between the engaged parties
**Ability to convene**	
	Negotiation
	Mediation
	Political and economic power mapping
	Collaborative group management, and problem-solving

### Formulating capacity development response

The capacity development response built on existing capacity assets to address the gaps identified in the capacity assessment [10]. This assessment was then used as the basis to develop the capacity development programme, which was approved by the Ministry in 2010, and then modified in 2011–2013 in response to an updated policy agenda and courses were added to support the newly prioritized health financing and purchasing reforms. The objectives of the capacity development programme were consistent with the objectives of the DSD and were informed by the various assessments. A number of different modalities were suggested for the capacity development programme in its improvement of competencies and skills, including short courses, study tours, mentoring and coaching, action learning, work placements and attachments and training over the medium- and long-term [11]. These modalities were selected on the basis of available resources (including time), the ‘fit’ for trainees and the skills or knowledge to be acquired, and the opportunities for interactive and problem-based learning they provide. The choice of modalities was also informed by the need to foster team-working and problem-solving in order to develop capacity for policy-making [11].

### Implementing capacity development response

Most of the teaching modalities recommended for the capacity building programme were implemented in 2010–2013. Seventeen modules on health policy and strategic planning, and financing and service purchasing were delivered by OPM (Oxford Policy Management) in the scope of projects, “Twinning Arrangement for Health Finance Capacity Building and Strengthening Strategic Purchasing project” and “DRG study tours for the Ministry of Health personnel in Switzerland,” supported by the World Bank and the Government of Kazakhstan [12]. Key implementation challenges included the disrupted continuity in national stakeholders (there were four changes of leadership in the DSD over a three year period and staff from the key Ministry of Health departments were also moved to affiliated organizations). This was a challenge encountered by other similar projects [13]. There was also a lack of engagement by staff in some departments. Although partners put significant effort into building an understanding of the process and benefits of the programme to their work and to them as individuals, it was not possible to fully change the attitude and behaviour (absenteeism) of selected individuals. Many staff argued that they did not have time to attend trainings, and they believed that there was a low probability that the capacity building programme would increase their career development opportunities.

### Evaluating capacity development response

The approach developed by Kaplan [14], was used to measure the effectiveness and cost-effectiveness of training activities as it is considered the human resources and training industry standard. Although an assessment using Kaplan’s approach is more complex, it was selected because it allows a comprehensive assessment from an organizational and not just individual perspective. This approach examines not only multiple aspects at the individual level, such as the satisfaction of trainees, the level of knowledge acquired, the application of new concepts or tools in daily work, but also the organizational benefits resulting from the changes in individual knowledge and practice; it seeks to understand how enhanced individual competencies may contribute to organizational performance. The approach requires that four aspects of the training be examined: reaction, learning, behaviour and results [15]. Reaction is the satisfaction of the trainees with the process and method of training; learning is the participants’ understanding of the content; behaviour is the application of the concepts or tools in daily work, as well as changes in behaviour and leadership style through discussion with senior managers. The results are then measured in terms of organizational benefits resulting from the training.

### Creating an enabling environment for career development

The Ministry of Health demonstrated both willingness and the ability to put skills nourished through the capacity building programme into effective use. The Minister urged the alignment of developed competencies with the wider organizational reforms and institutional changes. The promotion of the most distinguished trainees was proposed, including appointments to the position of the Vice-Minister and a Deputy Head of the Budget Department, but it is not clear that such meritocratic career development has been institutionalized within the Ministry of Health. This is an important point to note because increased capacity also increases the individual’s employability and mobility and it is important to ensure that transparent structures are in place to encourage the retention of staff [16]. Creating enabling environments also has other elements, such as ensuring that newly trained staff are given the opportunity to apply their new skills in their current roles; this has been identified as a particular challenge in other capacity building programmes in similar settings [17].

## Discussion and evaluation

One of the most challenging steps in the capacity development programme proved to be evaluating the capacity implementation response. Although the evaluation strategy was standard, and nominally accepted by the Ministry of Health, it did not appear possible to implement it fully in practice. The partial evaluation of the capacity development response was one of the weaknesses of the programme and future initiatives must overcome this weakness to develop stronger monitoring and evaluation. Nevertheless, the partial results from the evaluation did find improvements. These included the embedding of coaching and mentoring, which had become an integral part of the support from external partners throughout the three year programme. Knowledge technology transfer was reflected in joint assignments and deliverables produced by international and local consultants and ministry staff, and were highly valued by the Ministerial leadership [18, 19].

Behaviour change could only be assessed anecdotally. The extent to which the trained staff were applying the new concepts, approaches and tools in daily work, together with the progress made in professional behaviour and management and leadership style was observed. For example, there were marked improvements in the policy-making process through the establishing of working groups around technical topics which set up action plans, deadlines and clarified chains of responsibility; this fostered improvements in leadership style which became more cooperative and ‘democratic’ than previously. However, although these observations were continuous, they were not conducted in a structured way so do not reflect what was envisaged in the evaluation strategy.

On another hand, great emphasis was placed on understanding whether organizational benefits had resulted from the capacity building. Here, there were marked differences before and after the implementation of the training activities in policy development and strategic planning. This included better-planned, more timely and in-depth responses to tasks and assignments, increased self-confidence in dealing with daily work and improved quality of analytical reports, presentations and briefing notes as assessed by the staff themselves and observed by the consultants; and there was potential for promotion of staff members with notable engagement and learning. However, the best illustration of success was the high score given to the Health Sector Strategy for 2013–2017, as part of the Government’s annual assessment of all sector strategies. In previous years scores were lower, despite the fact that the Ministry was using external assistance in preparing the strategic documents. This time the strategy was developed by the DSD themselves, after completing training in policy-making and strategic planning. Thus, the results of the capacity building programme were reflected in institutional achievements.

This case study highlights the importance of strong political will and high level support for capacity building in ensuring the sustainability of programmes and ensuring sufficient resources are made available. Although it took time, it proved worthwhile to build a deep understanding of the goal, objectives and process of the capacity-building programme among all stakeholders. The capacity-building plan for both institutional and individual competence development also needed to be inclusive and rooted in thorough competence needs analyses.

However, it also shows that capacity-building programmes need to ensure full engagement with all local stakeholders, or where this is not possible, programmes need to be targeted more narrowly to those stakeholders who will benefit most, at the earliest stages for the greatest impact to be achieved. If some local stakeholders cannot see the benefit of participating (as detailed above), then it is unlikely they will benefit from the programme. Flexibility was also of key importance, so that the programme could be adjusted based on feedback from participants but also to ensure that the competencies taught could be and were used in the workplace. Feedback from participants was collected for some modules such as, for example, DRG coding trainings, where three aspects of the training were assessed by anonymous questionnaires which invited participants to assess: the relevance of the training to their needs, the quality of the training (i.e., was it practical, easy to understand, and participative), the applicability of training materials and knowledge obtained, etc. However, such written evaluations were not always possible because the participants generally saw them as a time consuming formality, and they preferred to give direct oral feedback on the relevance of the training programme and materials and the themes to be added. The programme was adjusted in light of this feedback in order to promote ownership.

It was important to assess existing capacity in the health sector and to stimulate engagement of local professionals and this has supported capacity development programmes in other areas which identified a number of trained individuals, working in both private and public sectors who are important assets for the country. This highlights the importance of also building the capacity of actors outside the health sector and even outside government. In parallel, the Ministry of Health capacity-building initiatives needed to be aligned with the broader capacity strengthening initiatives at the national level and integrated into the national education system. Nevertheless, capacity development is a perpetually evolving process of growth and positive change [6]. Despite significant investment in the capacity building programme, sustainability aspects were poorly addressed. This is a recognized challenge in the implementation of capacity building according to the UNDP framework [6]. To be effective, the programme needed to be more than a one-time initiative to guarantee its prolonged and multiplied effects, thus representing a ‘value added’ dimension of the capacity building initiative. The Ministry of Health needed to find ways to plan for comprehensive, long lasting capacity building efforts, with designated funding, and a clear accountability framework to monitor implementation.

## Conclusions

Kazakhstan has a challenging, but exciting, path forward to further develop a comprehensive and sustainable capacity-building approach for strengthening health professionals and organizations, by creating an enabling environment. The foundation laid by the Ministry of Health capacity-building programme for policy-making and strategic planning generates optimism for future achievements. The experience showed that complementary approaches to human resource development worked effectively in the context of organizations and systems, where an enabling environment was present, and country ownership and political will was complemented by strong technical assistance to design and deliver high quality tailor-made capacity building initiatives [20].
